# Omega-3 fatty acids in atherosclerosis and coronary artery disease

**DOI:** 10.4155/fsoa-2017-0067

**Published:** 2017-10-05

**Authors:** Magnus Bäck

**Affiliations:** 1 Divison of Valvular & Coronary Disease, Department of Medicine, Karolinska Institutet, Karolinska University Hospital & Center for Molecular Medicine, Stockholm, Sweden; 2 Nancy University Hospital, INSERM U1116 & University of Lorraine, Nancy, France

**Keywords:** docosahexanoic acid, eicosapentanoic acid, inflammation, lipoxygenase, resolvins

## Abstract

Omega-3 polyunsaturated fatty acids have emerged as possible protective factors associated with a decreased risk for myocardial infarction in populations with a high marine food intake, which may relate to effects on lipid metabolism, thrombosis and inflammation. Omega-3 fatty acids decrease triglyceride levels and also compete as substrates for enzymes involved in the biosynthesis of lipid mediators. The balance between omega-3-derived specialized proresolving mediators and pro-inflammatory lipid mediators from arachidonic acid metabolism can be measured as the resolvin-to-leukotriene ratio, which has been shown to predict subclinical atherosclerosis. The results of experimental, observational and randomized studies of omega-3 fatty acids are somewhat variable and should be interpreted in view of the models used and the populations studied.

## Lay abstract

Almost 40 years after the discovery of protective effects of fish oils on the heart and vessels, the mechanisms of action of omega-3 fatty acids remain unclear. In addition to effects on blood lipid levels, omega-3 fatty acids can be metabolized into bioactive lipid mediators. It has been proposed that such omega-3-derived bioactive lipids counteract the inflammation and blood clotting induced by lipid mediators from omega-6 fatty acids and that this contributes to the beneficial effects of fish oils.

Coronary artery disease (CAD) results from an underlying atherosclerosis, which is driven by an accumulation of lipids and low density lipoproteins (LDL) as well as the recruitment and activation of leukocytes in the vascular wall [1]. Lipid-laden macrophages, referred to as foam cells, make up a typical histological hallmark of the atherosclerotic chronic inflammation. Atherosclerotic plaques in the coronary artery progress over a long period of time and may be either asymptomatic or cause angina pectoris. Eventually, the atherosclerotic plaque may become unstable, leading to plaque rupture, platelet aggregation, thrombosis and coronary artery occlusion causing myocardial infarction. Despite a considerable improved prognosis for CAD patients as a result of prevention measures taken, there is still a significant residual risk in this patient group [1].

The omega-3 fatty acids docosahexanoic acid (DHA) and eicosapentanoic acid (EPA), have emerged as possible protective factors associated with a decreased cardiovascular risk in populations with a high marine food intake [2]. The potential protection offered by these omega-3 fatty acids in cardiovascular disease (CVD) may relate to their effects on lipid metabolism, thrombosis and inflammation as illustrated in Figure 1 and further discussed below. However, the results of experimental, observational and randomized studies of omega-3 fatty acids vary and have not consistently supported the use of omega-3 fatty acids in cardiovascular prevention.

**Figure 1.**
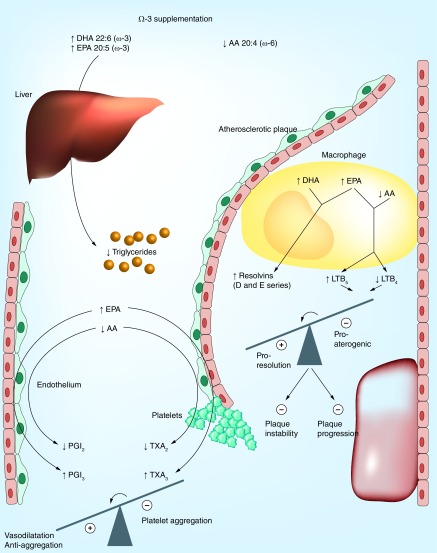
**Metabolic and vascular effects of omega-3 supplementation.** The circulating levels and cell membrane incorporation of the omega-3 fatty acids, DHA and eicosapentanoic acid (EPA) increase whereas the omega-6 fatty acid, arachidonic acid (AA) decreases. This change in fatty acid profile results in a reduced hepatic secretion of triglyceride-rich lipoproteins and lowering of hypertriglyceridemia. In the atherosclerotic lesion, infiltrating macrophages undergo a lipid mediator switch when omega-3 fatty acids and AA compete as substrates for the lipid mediator biosynthetic enzymes leading to the generation of, for example, the 5-series of leukotriene B (LT), which acts as inhibitors of pro-inflammatory LTB_4_ signaling. In addition, DHA and EPA serve as the substrate for, for example, the specialized proresolving mediators (SPMs) resolvins of the D- and E-series, respectively. In total, this lipid mediator switch will tip the balance away from inflammation toward proresolution, inhibited plaque progression and increased plaque stability. The competition of EPA and AA for the cyclo-oxygenase enzyme are shown in the bottom left part of the figure. Although EPA-derived thromboxane (TX) A_3_ from platelets exhibit weaker aggregatory actions compared with its AA-derived analog TXA_2_, the two prostacyclins, PGI_2_ and PGI_3_ are both anti-aggregatory and vasodilatory. Also the latter balance is hence tipped toward a beneficial profile by omega-3 supplementation. DHA: Docosahexanoic acid.

The aim of the present article is to appraise the potential mechanisms of action that provide the rationale for beneficial cardiovascular effects of the omega-3 fatty acids DHA and EPA, and to emphasize some of the points to consider when interpreting the results of studies in this field.

## Omega-3-induced effects on lipids

**Figure 2.**
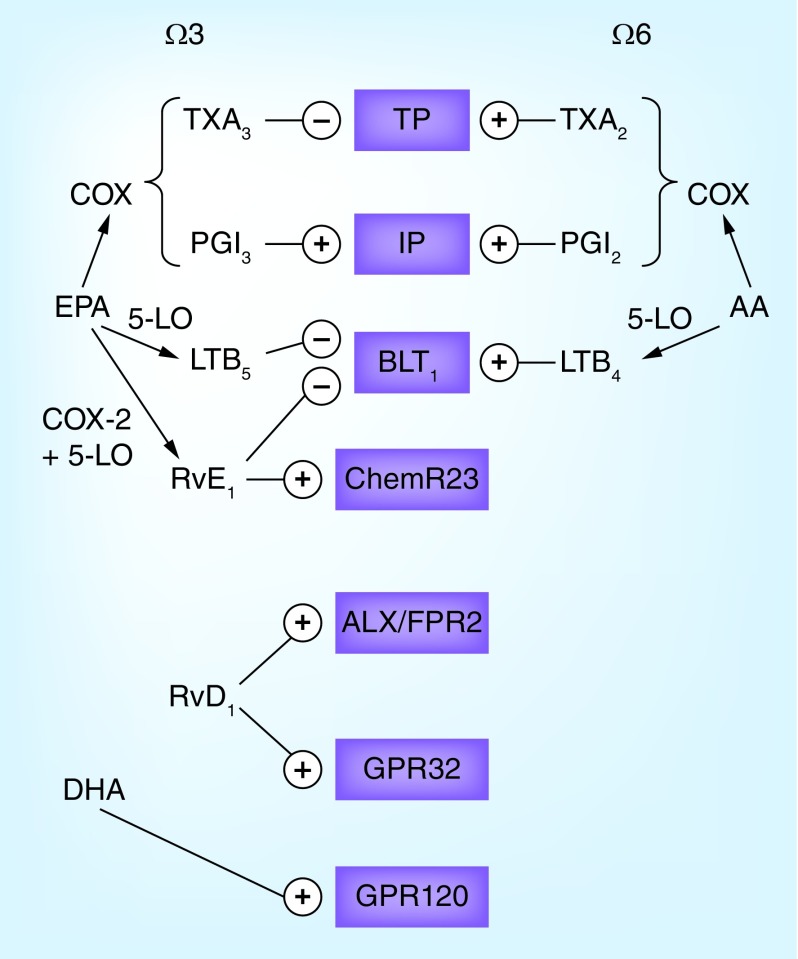
**Examples of the omega-3 and omega-6 metabolome and their receptors.** The cyclo-oxygenase metabolizes arachidonic acid (AA) into thromboxane (TX) A_2_ and prostaglandin I_2_ (PGI_2_, prostacyclin) and eicosapentanoic acid (EPA) into TXA_3_ and PGI_3_. Whereas TXA_3_ inhibits the effects of TXA_2_ on the thromboxane prostanoid (TP) receptor, PGI_3_ and PGI_2_ exhibit similar binding to the I prostanoid (IP) receptor. Similarly, 5-lipoxygenase (5-LO)-derived leukotriene (LT) B_4_ from AA and LTB4 from EPA exhibit opposing effects on the BLT receptor. Shown in the figure are also examples of specialized proresolving mediators, EPA-derived resolving (Rv) E1 and its receptor ChemR23 as well as DHA-derived RvD1 and its two receptors ALX/FPR2 and GPR32. Finally, the DHA receptor GPR120 is depicted.

Omega-3 fatty acids lower triglycerides by means of reduced hepatic secretion of triglyceride-rich lipoproteins [3]. A recent meta-analysis indicated that DHA supplementation appeared more efficacious compared with EPA in lowering triglycerides levels, but also revealed that DHA increased LDL cholesterol levels [4]. Those apparent differences depending on the omega-3 fatty acid used may be a result of, for example, differential effects of DHA and EPA on lipoprotein lipase activity, and that DHA may decrease LDL receptor levels [4,5]. DHA has also been identified as a ligand for the long chain free fatty acid receptor GPR120 (Figure 2) to directly transduce anti-inflammatory effects and beneficial metabolic profiles in experimental models [5].

## Omega-3-derived lipid mediators

### EPA-derived thromboxane & leukotrienes

In addition to altering lipid levels, omega-3 fatty acids also serve as the substrate for a group of bioactive lipid mediators formed from DHA and EPA [6]. For example, although AA metabolism by cyclo- and lipoxygenases yields pro-inflammatory prostaglandins/thromboxane and leukotrienes, respectively, the use of EPA as a substrate for these enzymes generates the same lipid mediators but with an extra double-bond. For example, the balance between thromboxane (TX) A_2_, a pro-aggregatory AA metabolite produced by platelets and prostaglandin I_2_ (PGI_2_, prostacyclin) is a key phenomenon in atherothrombosis. High omega-3 intake alters the relative ratios of the substrates competing for the cyclo-oxygenase enzyme leading to increased generation of EPA-derived TXA_3_ and PGI_3_ and decreased AA-derived TXA_2_ and PGI_2_ (Figure 1). Although TXA_3_ exhibits less aggregatory action compared with TXA_2_, the generated PGI_3_ has similar vasodilatory and anti-aggregatory actions as PGI_2_, providing one possible mechanism for how omega-3 fatty acids are associated with a beneficial thrombotic profile (Figure 1). The effects of these lipid mediators on their respective receptors are shown in Figure 2.

Also, leukotriene formation may be altered when EPA and AA compete as substrates for the 5-lipoxygenase (5-LO) enzyme (Figure 1). Indeed, the EPA-derived 5-LO product LTB_5_ is less biologically active compared with its AA-derived analog LTB_4_, but competes with LTB_4_ ligation at the BLT receptors (Figure 2) [7]. Several lines of evidence support LTB_4_ as an important pro-inflammatory macrophage-derived mediator and as such a driver of several steps in the atherosclerosis process [8]. The generation of 5-LO metabolites from EPA can hence be anticipated to act as an endogenous inhibitor of inflammation in atherosclerosis (Figure 1).

### Specialized proresolving mediators

More recently, specific enzymatic metabolism of DHA and EPA acids was discovered to yield a novel class of lipid mediators, which promote the resolution of inflammation [6]. This class of bioactive lipids has been named specialized proresolving mediators (SPM), and includes, for example, resolvins, marsins and protectins, which terminate the inflammatory response and promote return to tissue homeostasis to prevent the transition of acute into chronic inflammation [6]. This signaling is transduced through G-protein-coupled receptors. For example, the EPA-derived resolvin E1 (RvE1) activates the ChemR23 receptor and inhibits the BLT1 receptor (Figure 2) [7]. On the other hand, two receptors activated by the DHA-derived resolvin D1 have been identified, namely ALX/FPR2 and GPR32 (Figure 2) [7].

Furthermore, aspirin interaction with the SPM synthesis pathways stimulates the formation of more stable SPMs, which indicates that omega-3 fatty supplementation must be put in relation to aspirin use when interpreting the results of studies of omega-3 and cardiovascular risk. Importantly, recent data show that asprin-triggered proresolving omega-3 derivatives can be detected in CAD patients on concomitant aspirin and statin treatment [9]. It should however be pointed out that other studies failed to detect consistent plasma levels of SPMs after fish oil supplementation in healthy volunteers [10], and that there is currently some controversy around the formation of circulating SPMs [11]. Either urinary SPM metabolites [10] or saliva resolvin levels [12] may be an alternative approach to monitoring SPM formation in humans.

### Resolvin-to-leukotriene ratio

Since the omega-3-derived proresolving lipid mediators and pro-inflammatory thromboxane, prostaglandins and leukotrienes are formed in parallel during inflammation, the balance between these two opposing effects may be decisive if inflammation will either resolve or progress into a chronic phase. The applicability of the latter notion to CVD was recently reinforced by the observation that the salivary resolvin-to-leukotriene ratio predicted subclinical atherosclerosis [12]. Determining such resolvin-to-leukotriene ratios may hence be of importance not only for determining the relative role of omega-3 and -6-derived mediators but also as a novel marker of nonresolving vascular inflammation, which in turn may be of major importance for atherosclerosis progression (Figure 1).

## Experimental studies of omega-3 in atherosclerosis

Dietary supplementation with omega-3-rich fish oil in atherosclerosis-prone mice leads to increased incorporation of DHA and EPA in the aorta and the heart, whereas the AA content is decreased [13]. Despite somewhat variable results, the main conclusion, which emerges from the majority of the studies, is a beneficial effect of dietary omega-3 fatty acids on murine atherosclerosis [14]. In addition to this reduction in atherosclerotic lesion size, morphological characteristics of more stable lesions have also been observed [14]. In addition, fish oil supplementation prevents the rise in aortic levels of MMP-2 after exposure to chronic intermittent hypoxia [13], which could constitute one of the mechanisms behind a potential plaque-stabilizing effect of omega-3 supplementation (Figure 1).

Importantly, direct treatment of hyperlipidemic mice with SPMs (cf. *supra*) mimics the atheroprotective effects of omega-3 supplementation [15,16]. These recent studies reinforce the evidence that SPM formation and subsequent signaling by means of specific receptors constitute an important biological action of omega-3 fatty acids.

## Observational studies on dietary omega-3 & CV risk

The notion of the beneficial cardiovascular effects of omega-3 fatty acids originates from studies of Greenland inuits, among whom ‘death from cardiovascular disease is rare’. It was discovered that plasma and platelet levels of EPA and DHA were higher compared with other Scandinavian populations and inversely related to population rates of acute myocardial infarction [2]. Since this original observation almost 40 years ago, several observational diet-based studies and also dietary intervention studies have supported that fish intake appears to be protective for CVD [5], hence providing the rationale for the subsequent randomized controlled trials (RCT) of omega-3 supplementation.

## RCTs of omega-3 in CVD prevention

The results of the available RCTs on the effects of omega-3 supplementation on cardiovascular outcome [5] are variable, with both beneficial [17,18] and neutral [19,20] effects having been reported. It should however be pointed out that the study designs of omega-3 studies are heterogeneous, in terms of, for example, using omega-3 fatty acids for either secondary [17] or primary [20] prevention, as well as in mixed populations with and without known CAD and/or previous CV events [18,19]. The relatively low doses used and the variable ratio between EPA and DHA may also be implicated in the discrepancy of the available results.

## Current recommendations

Based on the results of the existing RCTs for omega-3 supplementation, it is today difficult to conclude on specific recommendations for CAD patients. In the 2016 guidelines on CVD prevention from the European Society of Cardiology, it is stated to be currently debatable whether omega-3 fatty acids exert a favorable effect on CVD [21]. In contrast, a recent consensus statement from the American Heart Association concluded that omega-3 supplements to patients with prevalent coronary heart disease (such as a recent myocardial infarction) is reasonable [22]. It is likely that the results of the currently ongoing trials of omega-3 supplementation will make it possible to make more firm recommendations in the future.

## Ongoing studies

Two large cardiovascular outcome studies of the effects of omega-3 fatty acids are currently being performed in subjects with hypertriglyceridemia and mixed dyslipidemia [5]. In the REDUCE-IT (NCT01492361), EPA ethyl ester (Vascepa^®^) is evaluated in combination with statin therapy against statin alone, whereas the STRENGTH (NCT02104817) study evaluates a combination of statin treatment with either carboxylic acids with a DHA-to-EPA ratio of 1:2.75 (Epanova^®^) or corn oil. It should be noted that the omega-3 doses used in the latter two studies (4 g/day) are higher compared with previous trials (1–1.8 g/day; [17–20]).

## Conclusion & future perspective

The mechanisms linking omega-3 fatty acid intake to beneficial cardiovascular effects currently require further exploration. The development of reproducible measures of SPM as biomarkers and indicators of omega-3 supplementation efficacy will facilitate to elucidate the contribution of EPA and DHA-derived lipid mediators to observed outcomes. Further characterization of the signaling pathways transduced by the ligation of the fatty acids and lipid mediators with their specific receptors will open up for more specific therapeutic stimulation of inflammation resolution in atherosclerosis. Optimization of formulations and doses for omega-3 supplementation may also be needed to enhance the efficacy on CVD. Ongoing trials of omega-3 supplementation will increase the clinical level of evidence in this field and strengthen future treatment recommendations for omega-3 fatty acids in coronary artery disease.

**Executive summary**

- Omega-3 fatty acids exert potent effects on both lipid metabolism and inflammation, two main determinants of the atherosclerosis process, as outlined in Figure 1.
- Based on the studies discussed in this Special Report, the following points may be of major importance to consider when interpreting the results of experimental, observational and randomized studies of omega-3 fatty acids, and their downstream lipid mediators:
  - The ratio of pro-inflammatory versus anti-inflammatory/proresolving mediators derived from different fatty acids may be decisive for an inflammatory response being either resolving or progressing to chronic inflammation [12].
  - The expression pattern of lipid mediator biosynthetic enzymes and specific receptors will determine downstream signaling by means of omega-3-derived lipid mediators [7,15].
  - Aspirin-triggered specialized proresolving mediators prevail in coronary artery disease patients on omega-3 supplementation [9].
  - The doses of omega-3 fatty acids used in currently ongoing studies in patients with hypertriglyceridemia are higher compared with previous randomized controlled trials on CV outcome.
  - The relative docosahexanoic acid and eicosapentanoic acid, content and formulation (ethyl esters, carboxylic acids) vary between different omega-3 formulations, and may differentially affect lipid metabolism, bioavailability and downstream lipid mediators.
